# Distribution and inflammatory potential of hepatitis C virus genotypes in the United States, 2011–2020

**DOI:** 10.1002/jgh3.70049

**Published:** 2024-11-04

**Authors:** Karthik Gnanapandithan, Lauren Stemboroski, Abbey Johnston, Maged P. Ghali

**Affiliations:** ^1^ Division of Hospital Internal Medicine Mayo Clinic Jacksonville Florida USA; ^2^ Division of Gastroenterology and Hepatology University of Florida Jacksonville Florida USA

**Keywords:** genotypes, hepatitis C, liver functions

## Abstract

HCV is marked by genetic diversity that impacts disease progression and outcome. Using the NHANES data from 266 HCV‐infected adults (2011–2020), this study infers that genotype 1a is the most prevalent (60.2%). Genotype 3 was associated with higher transaminase levels, though not statistically significant. These findings suggest a more aggressive phenotype for genotype 3. Despite pan‐genotypic treatment guidelines, this underscores the importance of continued HCV genotype surveillance and consideration for genotype‐specific treatment and monitoring strategies.

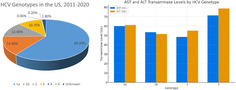

## Introduction

Despite advancements in antiviral therapies, hepatitis C virus (HCV) infection remains a significant public health challenge worldwide. Characterized by its high genetic variability, HCV is categorized into six major genotypes, each differing in epidemiological distribution, pathogenesis, and response to treatment. Understanding the prevalence and implications of these genotypes on liver function is crucial for optimizing management strategies and improving cure rates. With its nationally representative data, the National Health and Nutrition Examination Survey (NHANES) provides a unique resource to study these aspects of HCV within the United States.

HCV is divided into seven different genotypes and over 60 subtypes that vary in distribution worldwide. Globally, genotype 1 is the most prevalent, followed by genotype 3, while genotypes 4 and 5 are endemic in some lower socioeconomic countries.^1^ Based on reports from the Center for Disease Control and prior studies,^2^, ^3^ genotype 1 (Subtypes a and b) is responsible for about 75% of the HCV burden, genotypes 2 and 3 contribute 12–16% each, and the other genotypes less than 2% of the cases. Previous studies have documented differences in disease progression and treatment response across HCV genotypes, but this is not fully understood. The treatment guideline for HCV is moving toward a pan‐genotypic regimen, with less emphasis on genotype‐specific treatment, especially for treatment‐naive cases. The literature on the clinical differences between the different genotypes is limited. This study aims to throw light on the prevalence of HCV genotypes in the United States over 10 years from 2011 to 2020. We also look into the difference in liver function tests between them, which adds to the knowledge regarding their pathogenesis and hepatic inflammatory potential.

## Methods

The NHANES is an annual survey‐based database built with interviews, physical examinations, and laboratory data from the noninstitutionalized population of the United States. It began in 1960, but since 1999, it has been a highly structured annual survey. Around 5000 participants are selected annually based on a meticulous, multistage clustered sample design. Geographic and several demographic variables are considered, so that the results can be representative of and applicable to the US national population.^4^ Weights are formulated to account for this complicated sampling, nonresponse, and any adjustments after stratification to match the total population. Applying the weights during analyses of NHANES data ensures that the results can be representative of the US population. It is instrumental in studying chronic and infectious conditions, including their prevalence, trends, and risk factors. It has been a valuable resource for exploring the prevalence, distributions, and trends of hepatitis B and C infections in the United States.^5^, ^6^

We analyzed data from 266 adult participants aged 20 years and older with HCV infection enrolled in NHANES from 2011 to March 2020. Data collection after March 2020 was affected by the pandemic. Active infection was confirmed via positive HCV RNA. The VERSANT HCV Genotype 2.0 Assay was utilized for genotype determination. SAS 9.4 was used for statistical analysis, focusing on the weighted prevalence of HCV genotypes and comparing liver function test results across these genotypes using appropriate NHANES sample weights. All subjects provided written informed consent, and the NHANES study methods were approved by the ethics review board of the National Center for Health Statistics.

## Results

Genotype 1a is the most prevalent in the United States, comprising 60.2% of cases (Table 1). Genotype 1b was the second most common, with 14.5%. Genotypes 2 and 3 contributed to 12.4% and 10.7% of the HCV‐infected population, respectively. Individuals with genotype 3 exhibited higher average transaminase levels than the others. The mean aspartate transaminase (AST) and alanine transaminase (ALT) levels in those with genotype 3 were 71.3 (±8.1) U/L and 78.7 (±11.2) U/L. The next highest levels were those with genotype 1a, 59.9 (±3.2) U/L and 61.1 (±3.8) U/L, respectively. The differences in AST and ALT levels across genotypes did not reach statistical significance (*P* = 0.32 and 0.13). There was no difference in alkaline phosphatase, total bilirubin, and serum albumin levels. Table S1, Supporting information shows the gender distribution, body mass index, mean age, alcohol use, glycated hemoglobin, and lipid levels between the genotypes.

**Table 1:** Distribution of hepatitis C virus (HCV) genotypes in the US population and liver function tests (2011–2020)

HCV genotype (*n* = 266)	Number of subjects	Weighted % among HCV infected (SE)	AST (SE) (U/L)	ALT (SE) (U/L)	ALP (SE) (IU/L)	Total bilirubin (mg/dL)	Serum albumin (g/dL)
1a	172	60.2% (4.7)	59.9 (3.2)	61.1 (3.8)	80.1 (2.6)	0.64 (0.03)	4.07 (0.03)
1b	43	14.5% (2.2)	53.4 (7.6)	51.5 (7.5)	80.4 (2.8)	0.56 (0.05)	3.87 (0.09)
2	22	12.4% (3.4)	48.4 (4.7)	55.1 (6.2)	70.9 (2.4)	0.6 (0.07)	4.10 (0.07)
3	22	10.7% (3.0)	71.3 (8.1)	78.7 (11.2)	79.2 (2.8)	0.62 (0.09)	4.01 (0.08)
4	1	0.06% (0.06)	31 (0)	13 (0)	84 (0)	0.5 (0)	3.60 (0)
6	1	0.20% (0.19)	26 (0)	36 (0)	81 (0)	0.5 (0)	4.20 (0)
Unknown	5	1.8% (0.6)	50.8 (21.1)	47.0 (17.1)	70.1 (3.1)	0.41 (0.08)	3.90 (0.1)

ALP, alkaline phosphatase; ALT, alanine transaminase; AST, aspartate transaminase; SE, standard error.

## Discussion

Our study shows that genotype 1a has been the most prevalent from 2011 to 2020, followed by genotypes 1b, 2, and 3. A global study^7^ showed that in high‐income countries, 66% of the cases were genotype 1, 18% genotype 3, and 12% genotype 2. The genetic heterogeneity of HCV has been a major barrier to eliminating the infection and developing an effective vaccine. The different genotypes of HCV differ at about 30–35% of the nucleotide sites. Several DAAs targeting specific proteins are being developed and used as a combination regimen. Developing effective treatment strategies in different countries will require a thorough knowledge of the genotype distribution. This is likely to evolve with time based on differential treatment responses of different genotypes and with changes in immigration.

The pathogenic variability among HCV genotypes impacts treatment responses and clinical outcomes. Genotype 1 has been well‐documented for its initial poor response to interferon‐based therapies. However, it now achieves high cure rates with DAAs. In contrast, genotype 3, which is associated with more severe liver pathology, presents unique challenges. Traditionally, in patients with chronic HCV, steatosis is a well‐established factor associated with progression to cirrhosis, and other viral factors are considered less important.^8^ However, a meta‐analysis^9^ showed that there was a significantly faster progression toward fibrosis in those with genotype 3 than the others. It has been shown previously that genotype 3 is associated with a higher risk of steatosis, progression to fibrosis, and hepatocellular carcinoma.^10^ Previous studies^11^, ^12^ have also shown that genotype 3 is independently associated with lower rates of sustained virological response with DAA therapy than other genotypes. The mechanisms underlying these associations are not entirely understood but may involve differences in viral replication dynamics, interactions with host lipid metabolism, and immune responses. Genotype 3 has been shown to upregulate lipid droplet biogenesis in hepatocytes, facilitating viral assembly and promoting insulin resistance and oxidative stress, further exacerbating liver injury.^13^

A multinational study^14^ showed that a minimal monitoring approach, including no pretreatment genotyping, would be sufficient for most people undergoing DAA treatment for the first time. The recent IDSA/AASLD guidelines^15^ recommend a simplified approach with DAA without the need for genotyping for most treatment‐naive patients without decompensated cirrhosis. The higher levels of AST and ALT observed in patients with genotype 3 in our study, although not statistically significant, suggest a more aggressive disease phenotype with an increased inflammatory response and hepatocellular injury. This aligns with previous findings where genotype 3 infection was associated with rapid fibrosis progression and lower cure rates. Elevated transaminase levels are associated with a more rapid progression to cirrhosis in patients with HCV.^10^ Considering the disproportionate impact of genotype 3 on liver health, our results reinforce the importance of a genotype‐specific approach in managing HCV. Tailoring treatment and monitoring strategies to the unique challenges posed by genotype 3 could prove vital in reducing the progression to cirrhosis and the development of HCC.

The study has its limitations. NHANES excludes the institutionalized, incarcerated, and homeless population from its database. Although it is one of the largest databases for HCV‐related studies in the United States, the sample size was not large enough for stratified analysis. Data on the subjects' intravenous drug use status and HCV treatment status were unavailable.

In summary, the predominance of genotype 1a aligns with prior research but highlights the necessity for ongoing surveillance to monitor shifts in genotype distribution. The study confirms the variability in hepatic functions across HCV genotypes and the higher inflammation potential of genotype 3. While the current guidelines primarily focus on the degree of liver fibrosis, our study suggests that genotype‐specific nuances might also dictate treatment regimen, duration, and follow‐up. Future research should aim to explore these relationships in larger, longitudinal cohorts to better titrate treatment paradigms and improve outcomes in patients with HCV.

## Supporting information


**Table S1.** Demographics and laboratory values of the different genotypes (2011–2020).
