# Supplementary material for: Distribution and inflammatory potential of hepatitis C virus genotypes in the United States, 2011–2020
Source: JGH Open. 2024 Nov 4;8(11):e70049. doi: 10.1002/jgh3.70049 (PMC11533706; doi:10.1002/jgh3.70049)
Supplement: Supplementary file 1 — Table S1. Demographics and laboratory values of the different genotypes (2011–2020). [file JGH3-8-e70049-s001.docx]

**Table S1: Demographics and laboratory values of the different genotypes (2011-2020)**

| **HCV Genotype**  **(n=266)** | **Number of subjects** | **Age (SE)** | **Sex**  **Males%** | **BMI (SE)** | **Alcohol use*** | **A1c (SE)** | **TG** (SE)**  **(mg/dL)** | **LDL** (SE)**  **(g/dL)** |
| --- | --- | --- | --- | --- | --- | --- | --- | --- |
| 1a | 172 | 52.5 (1.1) | 69.1% | 27.2 (0.5) | 33.2% | 5.8 (0.1) | 111.5 (9.8) | 106.2 (3.9) |
| 1b | 43 | 60.8 (1.9) | 72.1% | 30.1 (1.6) | 36.3% | 6.2 (0.3) | 129.4 (22.3) | 109.4 (4.3) |
| 2 | 22 | 54.1 (2.4) | 83.2% | 31.8 (1.7) | 34.1% | 5.6 (0.1) | 141.6 (28.4) | 108.7 (5.3) |
| 3 | 22 | 52.4 (1.5) | 63.0% | 24.0 (0.7) | 31.8% | 5.5 (0.05) | 106.3 (8.5) | 96.7 (9.7) |
| 4 | 1 | 62 | - | 22.1 | - | 4.6 | - | - |
| 6 | 1 | 44 | - | 28.4 | - | 5.7 | - | - |
| Unknown | 5 | 49.8 (2.7) | 48.6% | 29.3 (1.4) | 41.1% | 5.3 (0.1) | 111.7 (16.4) | 99.5 (3.7) |

BMI: Body Mass Index, TG: Triglycerides, LDL: Low-Density Lipoprotein

*Subjects reporting drinking alcohol 3 or more times a week were considered.

**26% of data was unavailable; **61% was unavailable: Multiple imputation analyses were used in both these cases.*
